# A Perspective on the Link between Mitochondria-Associated Membranes (MAMs) and Lipid Droplets Metabolism in Neurodegenerative Diseases

**DOI:** 10.3390/biology12030414

**Published:** 2023-03-08

**Authors:** Tânia Fernandes, M. Rosário Domingues, Paula I. Moreira, Cláudia F. Pereira

**Affiliations:** 1 CNC—Center for Neuroscience and Cell Biology, CIBB—Center for Innovative Biomedicine and Biotechnology, University of Coimbra, 3004-504 Coimbra, Portugal; 2 IIIUC—Institute for Interdisciplinary Research, University of Coimbra, 3030-789 Coimbra, Portugal; 3 CACC—Clinical Academic Center of Coimbra, 3004-561 Coimbra, Portugal; 4 Faculty of Medicine, University of Coimbra, 3000-548 Coimbra, Portugal; 5 Mass Spectrometry Centre, Department of Chemistry & CESAM, Department of Chemistry & LAQV-REQUIMTE, Campus Universitário de Santiago, University of Aveiro, 3810-193 Aveiro, Portugal

**Keywords:** neurodegenerative disorders, ER–mitochondria contacts, lipid storage, lipophagy, energy production

## Abstract

**Simple Summary:**

Neurodegenerative diseases affect millions of people worldwide, and their prevalence rises dramatically with age. Although a few therapeutic options help to relieve symptoms associated with these disorders, slowing their progression is not presently possible and no cure exists. Therefore, the scientific community continues to improve our understanding regarding alterations in cellular function and develop new approaches for prevention and treatment. In recent years, the endoplasmic reticulum–mitochondria interaction, called mitochondria-associated membranes, has gained special attention in neurodegenerative diseases since this region is involved in regulation of several cellular processes, including lipid synthesis and transport, and is affected by defective communication between both organelles. Here, we provide an overview of the structure and function of mitochondria-associated membranes and how these structures are involved in metabolism of lipids, especially in biogenesis and degradation of lipid droplets in neurodegenerative disorders. These lipid storage organelles prevent lipotoxicity and maintain energy homeostasis, and changes in their metabolism can accelerate cellular stress, contributing to development of neurodegenerative diseases. This review supports the growing research interest in lipid (dys)metabolism as a key player in neurodegeneration.

**Abstract:**

Mitochondria interact with the endoplasmic reticulum (ER) through contacts called mitochondria-associated membranes (MAMs), which control several processes, such as the ER stress response, mitochondrial and ER dynamics, inflammation, apoptosis, and autophagy. MAMs represent an important platform for transport of non-vesicular phospholipids and cholesterol. Therefore, this region is highly enriched in proteins involved in lipid metabolism, including the enzymes that catalyze esterification of cholesterol into cholesteryl esters (CE) and synthesis of triacylglycerols (TAG) from fatty acids (FAs), which are then stored in lipid droplets (LDs). LDs, through contact with other organelles, prevent the toxic consequences of accumulation of unesterified (free) lipids, including lipotoxicity and oxidative stress, and serve as lipid reservoirs that can be used under multiple metabolic and physiological conditions. The LDs break down by autophagy releases of stored lipids for energy production and synthesis of membrane components and other macromolecules. Pathological lipid deposition and autophagy disruption have both been reported to occur in several neurodegenerative diseases, supporting that lipid metabolism alterations are major players in neurodegeneration. In this review, we discuss the current understanding of MAMs structure and function, focusing on their roles in lipid metabolism and the importance of autophagy in LDs metabolism, as well as the changes that occur in neurogenerative diseases.

## 1. Introduction

Mitochondria can communicate with the endoplasmic reticulum (ER) through biochemical and physical contact sites with an intermembrane distance of about 10 to 80 nm [1,2,3]. These dynamic structures, referred to as mitochondrial-associated membranes (MAMs), are enriched in proteins and lipids and regulate several cellular functions, including lipid synthesis and trafficking, Ca^2+^ signaling, autophagy, mitochondrial and ER dynamics, the ER-stress-induced unfolded protein response (UPR), redox status, inflammation, and apoptosis [4,5,6,7]. Since MAMs are an essential platform for non-vesicular transport of phospholipids and cholesterol between the ER and mitochondria [8,9], this region is enriched in proteins involved in lipid metabolism, including lipid droplets (LDs) formation [10,11]. These storage organelles are highly dynamic and can interact with other organelles, including mitochondria [12,13]. LDs function as fatty reservoirs for energy production or membrane biogenesis and store excess of lipids, preventing accumulation of unesterified lipids and, consequently, lipotoxicity and oxidative stress [14,15,16]. Breakdown of the LDs core by lipolysis or lipophagy can provide energy for metabolic processes and membrane biosynthesis during nutrient deprivation or cell growth, which requires membrane expansion and high rates of phospholipid biosynthesis [17]. The neutral lipids are converted to FAs and delivered to mitochondria for energy production via β-oxidation and the Krebs cycle [12]. Furthermore, autophagy can protect cells by promoting degradation of misfolded and aggregated proteins, dysfunctional mitochondria, and LDs; however, alterations in this process can lead to premature aging, tissue degeneration, and proinflammatory responses [18]. The brain is the second most lipid-rich organ and changes in the lipid composition of the central nervous system (CNS) affect cell functioning and normal neural activity [19,20]. Furthermore, in recent years, disruption of the autophagic process and pathological lipid deposition have been described as hallmarks of several neurodegenerative diseases, including Alzheimer disease (AD), amyotrophic lateral sclerosis (ALS), and Parkinson’s disease (PD) [18,21]. Increased lipid accumulation can enhance FAs oxidation rate and reactive oxygen species (ROS) production, potentiating oxidative stress, and can lead to mitochondrial damage, lysosomal dysfunction, defective autophagy, and activation of inflammatory responses [22,23].

In this review, we provide an overview of MAMs structure and functions and discuss the involvement of these structures in lipid metabolism, as well as the importance of LDs biogenesis, lipolysis, and lipophagy-mediated degradation in neurodegenerative disorders.

## 2. Mitochondria-Associated Membranes (MAMs)

The physical interaction between the ER and mitochondria was observed for the first time in 1969 in mammalian ovary tissue by electron microscopy [24]. However, only 20 years later were the ER–mitochondria contacts isolated from rat liver tissue through subcellular fractionation using a Percoll density gradient [25]. The physical contacts between these two organelles that were called mitochondria-associated ER membranes (MAMs) [26] are extremely flexible and recruit specific proteins to modulate their formation and function in response to the metabolic state of the cell [2,8]. For example, when nutrients drop in hepatocytes, the distance between the ER and mitochondria, as well as mitochondrial surface in contact with the ER, increase [27]. MAMs enable crosstalk between both organelles, which impact several cellular processes, such as Ca^2+^ signaling, energy and lipid metabolism, mitochondrial and ER dynamics, ER stress responses, inflammation, apoptosis, and autophagy [5,15,26,28]. The interaction between the ER and mitochondria is mediated by several proteins present in the ER membrane and in the outer mitochondrial membrane (OMM). ER–mitochondria tethering is formed by: (1) ER 1,4,5-triphosphate receptor (IP3R), chaperone 75-kDa glucose-regulated protein (Grp75), and voltage-dependent anion-selective channel (VDAC) in OMM [29]; (2) vesicle-associated membrane-protein-associated protein B (VAPB) in the ER and mitochondrial protein tyrosine phosphatase interacting protein 51 (PTPIP51) [30]; (3) ER B-cell-receptor-associated protein 31 (BAP31) and fission 1 protein (FIS1) in mitochondria [31]; (4) mitofusin 2 (MFN2) in the ER and MFN2 or mitofusin 1 (MFN1) present in the OMM [32].

MAMs are microdomains specialized in Ca^2+^ transfer from the ER to mitochondria. The ER Ca^2+^ release by the IP3R increases the cytoplasmatic Ca^2+^ levels at the ER–mitochondria contact sites, leading to mitochondrial Ca^2+^ import [33]. The ER sigma-1 receptor (Sigma1R) is localized at MAMs and interacts with the chaperone binding immunoglobulin protein (BiP) to stabilize the IP3R that prolongs Ca^2+^ signaling between the organelles [34,35]. The Ca^2+^ ions enter mitochondria through VDAC and the mitochondrial calcium uniporter (MCU) located in the OMM and in the inner mitochondrial membrane (IMM), respectively, and accumulate in the mitochondrial matrix [29,36]. Protein GRP75 allows flux of Ca^2+^ at MAMs by enabling binding between ER IP3R and mitochondrial VDAC [8,15]. Thus, MAMs regulate mitochondrial Ca^2+^ levels and play an important role in several mitochondrial functions, such as oxidative phosphorylation, ROS generation, and signaling [15]. In addition, Ca^2+^ transfer between the ER and mitochondria regulates apoptosis since excessive accumulation of Ca^2+^ in the mitochondrial matrix can induce release of pro-apoptotic factors, such as cytochrome c, into the cytosol due to opening of mitochondrial permeability transition pore (mPTP) [1,8].

In addition, to participate in transfer of Ca^2+^ from the ER to mitochondria, MAMs play an essential role in intracellular lipid metabolism as a place of non-vesicular lipid exchange between both organelles [15]. The enzymes involved in lipid trafficking and synthesis are present in MAMs, including the ACAT1/SOAT1 that catalyzes esterification of cholesterol into CE [10], the DGAT2 that catalyzes the final step of TAG synthesis and LDs formation [11], phosphatidylserine synthases 1 and 2 (PSS1 and PSS2) and phosphatidylethanolamine N-methyltransferase 2 (PEMT2) that are involved in phospholipid synthesis [37,38], and fatty acid CoA ligase 4 (FACL4/ACSL4) that activates long-chain FAs, which will be used for synthesis of complex lipids or acylated proteins [39].

Synthesis of phospholipids usually occurs in the ER, and phospholipids are then translocated to other organelles’ membranes, including to mitochondria. MAMs-resident enzymes PSS1 and PSS2 convert phosphatidylcholine (PC) and phosphatidylethanolamine (PE), respectively, into phosphatidylserine (PS) in the ER. After translocation to mitochondria, PS is converted into PE by PS decarboxylase (PSD) and PE synthetized in mitochondria is imported by the ER, where it is methylated by PEMT2 and converted into PC [5,8,9,40,41,42]. Mitochondria interact with both smooth ER and rough ER at a fixed distance that varies between ~10–25 nm and ~50–80 nm, respectively, and the contacts extend over hundreds nm in length [2]. The number, thickness, and extension of MAMs are regulated parameters of the cells and determine the activity of this subcellular compartment [2]. Depending on the metabolic state of the cell, the surface of mitochondria in apposition to ER change, for example, in HeLa cells, ranges from 5% to 20% [43] and in mouse liver cells varies between 4% and 11% [27]. Furthermore, width of MAMs depends on the metabolic state and influence the cellular processes that occur in this region. The distance of ~12–24 nm between the ER and mitochondria supports higher rates of Ca^2+^ transfer because assembly of the IP3R-GRP75-VDAC complex is favored [44,45,46]. Instead, a width of 10 nm enables assembly of proteins that mediate lipid transfer, which requires tunneling of a lipid through hydrophobic channels formed by proteins present in both organelles’ membranes [47]. Moreover, it was observed in fibroblasts from AD patients more “long” (50–200 nm) and “very-long” (>200 nm) MAMs accompanied by increased lipid biosynthesis/transfer between the ER and mitochondria [48]. This suggests that an increase in the length of the ER that interacts with the mitochondrial surface promotes transference of lipids between both organelles and leads to lipid dyshomeostasis in pathological conditions.

**Figure 1:**
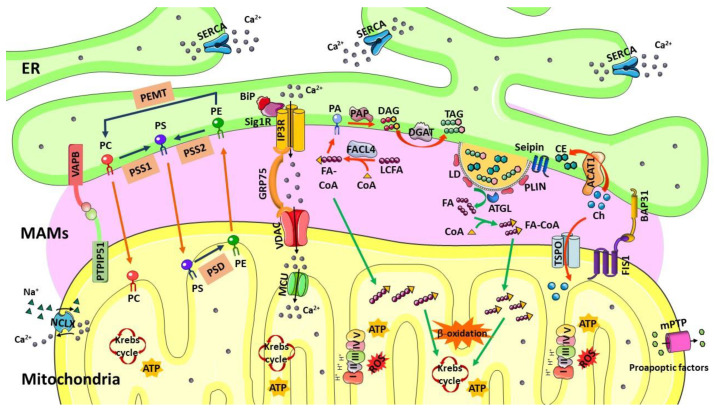
ER–mitochondria tethering and lipid metabolism. Physical interaction between ER and mitochondria is called mitochondria-associated membranes (MAMs) and is formed by several protein complexes, including VAPB-PTPI51, IP3R-GRP75-VDAC, and BAP31-FIS1. This region regulates several cellular functions, such as Ca^2+^ signaling and lipid synthesis and trafficking. Ca^2+^ released from the ER through IP3R is transported to mitochondria by VDAC and MCU present in OMM and IMM, respectively. GRP75 acts as a linker between IP3R and VDAC and Sig1R interacts with chaperone BiP to stabilize IP3R. Furthermore, MAMs are enriched in proteins involved in lipid metabolism and play an important role in phospholipid transfer from the ER to mitochondria. Phospholipid PC synthetized in the ER can be directly translocated to mitochondria or converted into PS by PSS1 at the ER; then, PS is imported by mitochondria and converted to PE by PSD. The PE synthesized in mitochondria can be transported to the ER and converted to PC or PS by PEMT or PSS2, respectively. At MAMs, protein FACL4 binds CoA to long-chain FAs before fatty acyl-CoA is transported to mitochondria for β-oxidation or used as a precursor for synthesis of PA, which is then converted in DAG. DGAT2 catalyzes synthesis of TAG from DAG, and protein ACAT1 catalyzes synthesis of Ch in CE, which are stored in LDs. Seipin facilitates growth of nascent LDs that are surrounded by PLINs proteins. Lipids stored in LDs can be converted to FA by lipolysis and used for β-oxidation to produce ATP. Cholesterol can also be transported from ER to mitochondria through TSPO protein.

The enzymes involved in cholesterol biosynthesis are also present in MAMs, such as the ACAT1 that catalyzes the conversion of free cholesterol into CE, which contributes to maintain equilibrium between membrane-bound and cytoplasmic LDs-stored cholesterol [8]. Under stress conditions, cholesterol transport to mitochondria is maintained, and steroidogenesis is initiated by cytochrome P450 [49]. MAMs also contain enzymes involved in ceramides’ biosynthesis, including sphingomyelin phosphodiesterase (SMase), ceramide synthase (CerS), and dihydroceramide desaturase (DES) [50,51,52]. Cholesterol depletion and inhibition of de novo synthesis of ceramides cause in CHO cells relocation of MAMs proteins, such as the Sigma1R and the IP3R, towards the ER cisternae, demonstrating that alterations in synthesis of cholesterol and ceramides at MAMs affect the structure of these contact sites [53]. Furthermore, it was observed that a decrease in cholesterol levels in MAMs promotes interaction between the ER and mitochondria and changes lipid synthesis, as shown by the reduction in de novo PS synthesis with simultaneous increase in PE synthesis [49] (Figure 1).

MAMs have a unique lipid composition that resembles lipid rafts [54], with an accumulation of cholesterol and glycosphingolipids that can be required for MAMs formation [53,54,55]. The lipid rafts homeostasis is regulated by SMase and phospholipase enzymes present at MAMs [50,56], which provide a platform for intracellular trafficking and signal transduction, particularly within neurons. The mechanisms of synthesis and hydrolysis of sphingomyelin are important for lipid rafts homeostasis [57] since sphingomyelin interacts with cholesterol and provides structural support for these microdomains [58]. Consequently, alterations in SMase enzymes have been associated with neurodegeneration [59,60]. Furthermore, the ER lipid-raft-associated protein 1/2 (ERLIN1/2) localized at MAMs’ lipid-raft-like microdomains [61] are involved in cholesterol homeostasis through regulation of sterol-regulatory-element-binding proteins (SREBPs), which include SREBP cleavage-activating protein (Scap) and insulin-induced gene 1 (Insig 1) [62]. Under cholesterol depletion conditions, the SREBP-Scap binds to COPII-coated vesicles for subsequent transport to Golgi and activates cholesterol and FAs biosynthesis [62]. However, under high cholesterol levels, ERLINs suppress cholesterol production by stabilizing the SREBP–Scap–Insig complex at the ER [63]. Furthermore, these lipid microdomains can also play a key role in recruitment of autophagy-related molecules to MAMs and promote formation of autophagosomes [54,64]. Therefore, lipid rafts dysregulation affects neurotrophic signaling and leads to apoptotic neuronal death.

Abbreviations: ACAT1, acyl-CoA cholesterol acyltransferase; ATGL, adipose triglyceride lipase; BAP31, B-cell-receptor-associated protein 31; BiP, binding immunoglobulin protein; Ch, cholesterol; CE, cholesteryl esters; CoA, coenzyme A; DAG, diacylglycerol; DGAT, diacylglycerol O-acyltransferase; ER, endoplasmic reticulum; FA, fatty acid; FACL4, fatty acid CoA ligase 4; FA-CoA, fatty acyl CoA; FIS1, mitochondrial fission 1; GRP75, glucose regulated protein 75; IP3R, inositol 1,4,5 trisphosphate receptor; LCFA, long chain fatty acid; LD, lipid droplets; MAMs, mitochondria-associated membranes; MCU, mitochondrial calcium uniporter protein; mPTP, mitochondrial permeability transition pore; NCLX, mitochondrial sodium/calcium exchanger protein; PA, phosphatidic acid; PAP, PA phosphatase; PC, phosphatidylcholine; PE, phosphatidylethalonamine; PEMT, PE N-methyltransferase; PSS1/2, PLIN, perilipin; PS, phosphatidylserine; PSS1/2, PS synthases 1/2; PSD, PS decarboxylase; PTPIP51, protein tyrosine phosphatase interacting protein 51; TAG, triacylglycerol; TSPO, translocator protein; SERCA, sarco/endoplasmic reticulum Ca^2+^ ATPase; Sig1R, sigma 1 receptor; VAPB, vesicle-associated membrane-protein-associated protein B; VDAC, voltage-dependent anion channel.

## 3. Lipid Droplets and Lipid Metabolism at MAMs

Lipids are important building blocks of cellular membranes and signaling mediators, and FAs are essential for energy production and storage [17]. However, overaccumulation of essential intermediate lipids, such as DAG, FAs, and ceramides, becomes toxic, leads to lipid stress, and triggers cell death through activation of several signaling pathways, namely the UPR [15,65]. Thus, cells store the excess of lipids in LDs to prevent lipotoxicity and oxidative stress once lipid accumulation can induce ROS generation and ER stress [66,67] and maintain energy homeostasis [14,15]. Qin et al. observed that high concentrations of free FAs (FFAs) induces lipid overload and oxidative stress in L-02 cells [68]. Furthermore, it was also observed that TAG accumulation and storage in LDs protects CHO and 25RA cells against FAs-induced lipotoxicity [69]. In mouse embryonic fibroblasts (MEFs) derived from Dgat1^−/−^ mice, decreased TAG levels were detected in response to FAs supplementation together with cell death prevention, indicating that disruption in the FFAs to TAG conversion plays a critical role in protection against lipotoxicity [69]. Bosma et al. also observed that the storage of lipids in LDs is protective against palmitate-induced ER stress in human cardiomyocyte cell line AC16, derived from primary cultures of adult ventricular heart tissue [70].

LDs are dynamic lipid storage organelles that present a core of neutral lipids, most commonly TAG and CE, surrounded by a phospholipid monolayer with integral and peripheric associated regulatory proteins, which are involved in structural, regulatory, or enzymatic functions [12,71,72]. LDs’ biogenesis starts with esterification of neutral lipids. Enzyme FACL/ACSL catalyzes formation of a thioester bond between the long FAs and coenzyme A, synthesizing long-chain fatty acyl-CoA that can be transported to mitochondria for β-oxidation or used as a precursor for synthesis of new phospholipids, such as phosphatidic acid (PA) [73,74,75]. Then, PA is converted to DAG by PA phosphatase (PAP)/Lipin and DGAT1 and DGAT2 catalyzes synthesis of TAG from DAG. Furthermore, sterols (such as cholesterol) are converted to CE by ACAT1 and ACAT2. TAG and CE are synthesized between the leaflets of the ER bilayer and stored in LDs [76,77,78]. After the synthesis of neutral lipids, the ER membrane protein seipin and other factors involved in LDs formation are recruited to the lens structure and facilitate growth of the nascent LDs [76,77]. Several studies correlate LDs formation with MAMs. Area-Gomez et al. confirmed that the ACAT protein is more abundant in the MAMs fraction and has ~3-fold higher enzymatic activity when compared to the ER and mitochondria fractions [48]. DGAT2 was also found localized at the MAMs fraction, but this protein can also associate with mitochondria as a peripheral protein. In COS-7 cells treated with oleic acid to induce TAG synthesis and, consequently, LDs formation, DGAT2 locates near the LDs surface, where it co-localized with mitochondria [79]. These results suggest that dynamic interaction between LDs, MAMs, and mitochondria is important during active lipid synthesis [79]. Recently, it was also reported that seipin is enriched at MAMs in adipocytes, where it plays an important role in mitochondrial Ca^2+^ import and metabolism. The authors demonstrated that seipin deficiency affects the flux of Ca^2+^ into mitochondria and decreases ATP production in the 3T3-L1 adipocytes cell line, and that inducible deletion of seipin from adipocytes in vivo causes early mitochondrial dysfunction and compromises lipid metabolism [80]. The fact that MAMs are enriched in proteins involved in LDs formation shows the importance of this sub-cellular region in this process. Furthermore, during conditions that promote very active LDs expansion, de novo phospholipids synthesis and trafficking, which are regulated by MAMs, are required to maintain homeostasis [17]. Last, in some mammalian cells, LDs bud from the ER membrane and grow through fusion with other LDs or local lipid synthesis in cytoplasm [17]. However, LDs need to tightly interact with the ER to coordinate lipid storage and formation of cellular membranes [81]. Re-localization of TAG synthesis enzymes from the ER to the LDs surface provides direct synthesis of TAG from cellular lipidic sources [82], including FA derived from autophagic breakdown of phospholipids [12]. Furthermore, LDs also incorporate cell debris to provide protection against lipotoxicity during periods of stress and starvation [81].

LDs are relevant players in membrane and lipid trafficking, ER response to stress, protein storage and degradation, infection, and immunity [83]. However, the main function of LDs is thought to be fatty reservoirs for energy production or membrane biogenesis for many cell types and can also protect from FAs toxicity [16]. These dynamic organelles change between periods of growth and consumption through enzymatic hydrolysis mediated by lipases (lipolysis) or selective autophagy (lipophagy) [17]. The lipolysis process involves several cytoplasmic lipases, including adipose triglyceride lipase (ATGL), hormone-sensitive lipase, and monoacylglycerol lipase, which catalyze hydrolysis of TAG and CE [84]. Subsequently, lipophagy can target LDs for degradation and release of FFAs and glycerol [85].

During starvation, lipids stored on LDs are mobilized for energy production or phospholipids synthesis [17]. The neutral lipid lipases, such as PNPLA2 (patatin-like phospholipase containing 2)/ATGL, mediate breakdown of LDs, releasing FAs from stored TAG for energy production in mitochondria via β-oxidation and the Krebs cycle [12]. Under prolonged starvation, degradation of organelle membranes by autophagy releases FAs that are packaged in new LDs before being transferred to mitochondria through LDs–mitochondria contacts [12], where the proximity of the membranes is 10–30 nm [13]. Therefore, the interaction between LDs and mitochondria increases in response to nutrient starvation [12,81,86]. Furthermore, the LDs–mitochondria interaction can decrease the cytosolic content of FAs, preventing lipotoxicity [12,81]. The extensive contacts between LDs and other organelles, including the ER, mitochondria, lysosomes, and peroxisomes, probably facilitates interorganelle transfer of lipids and reduces levels of cytosolic FFAs, which avoids lipotoxicity and/or abnormal lipid signaling [12,17,81]. However, a study performed in brown adipocytes showed that, in mitochondria associated with LDs, the β-oxidation is impaired and synthesis of ATP is stimulated. Probably, these mitochondria support growth and expansion of associated LDs by supplying ATP for FAs activation required for TAG synthesis. This suggests that LDs–mitochondria contacts could be used for both lipogenesis and lipolysis in different cells or under different metabolic conditions [87].

Interaction between LDs and mitochondria enables direct transport of lipids to mitochondria for β-oxidation and energy production [81,86,88]. On the other hand, mitochondria provide ATP and nicotinamide adenine dinucleotide phosphate (NADPH) to support synthesis of FAs, TAG, and glycolytic precursors from the Krebs cycle for esterification of FAs into TAG in the ER and for storage of TAG in LDs [87,89]. This suggests that mitochondria not only contribute to LDs breakdown but also LDs formation [87,89] and that the ER–mitochondria contact is essential because it allows the LDs to interact with mitochondria because LDs’ biogenesis occurs at the ER membrane [90,91] (Figure 1). Recently, it was reported that OMM’s protein mitoguardin 2 (MIGA2) connects mitochondria to the ER and LDs via the ER membrane’s VAPA or VAPB, promoting TAG synthesis from non-lipidic precursors [89]. Overexpression of *Miga2* in fly increases formation of ER–mitochondria contacts and causes severe neurodegeneration [92]. Furthermore, overexpression of LDs-resident protein perilipin 5 (PLIN5), which is highly expressed in oxidative tissue (liver, skeletal muscle, heart, and brown adipose tissue) also induced recruitment of mitochondria into LDs clusters and mediated LDs–mitochondria tethering [87,93]. Boutant, et al. also found in brown adipocytes that MFN2 directly interacts with PLIN1, facilitating contacts between LDs and mitochondria and promoting coupling of TAG hydrolysis with FA oxidation after adrenergic stimulation. Moreover, knockout of *Mfn2* decreased LDs–mitochondria contacts as well as leads to respiratory capacity impairment and a blunted response to adrenergic stimuli in brown adipocytes [94].

Inhibition of the DGAT1 and DGAT2 enzymes in astrocytes prevents de novo LDs’ biosynthesis, decreasing the number of cells by ~40%, which indicates that the turnover of LDs in resting astrocytes is important for maintenance of cell proliferation and/or survival [95]. In primary cortical astrocytes, inhibition of DGAT-suppressed oleic acid-induced LDs accumulation, suggesting that extracellular FFAs are stored in LDs to protect the cells from the lipotoxic stress [96]. Moreover, in non-adipocyte cells, the excessive accumulation of FFAs can induce oxidative and ER stress due to changes in an organelle’s membrane structure and function, leading to production of toxic metabolites and activation of death signaling pathways. However, storage of excess FFAs in LDs prevents lipotoxic cell death and represents a cytoprotective strategy [90]. In *Drosophila* neural stem cells, it was shown that relocation of membrane phospholipids to lipid droplets TAG, especially lipid species with polyunsaturated FAs (PUFAs) that are more susceptible to ROS-mediated oxidation, protects PUFAs from peroxidation [97].These results show that sequestration of FFAs as TAG and cholesterol as CE inside LDs prevents its deleterious effects once these lipids can act as detergents that disrupt the membrane integrity or, at higher levels, can be integrated into cytotoxic species, such as ceramide, acylcarnitine, and DAG, protecting the cells from lipotoxicity [17]. However, catabolism of LDs into FFAs by lipolysis and autophagy is an essential cellular pathway required for energy production in the form of ATP and provides metabolic energy for several cellular processes, including membrane synthesis and molecular signaling [98].

## 4. MAMs and Lipid Droplets Degradation

The FAs present as TAG in the core of the LDs can be mobilized by lipolysis or lipophagy to provide energy for metabolic processes and membrane biosynthesis during periods of nutrient deprivation or cell growth, which requires membrane expansion and high phospholipids’ biosynthesis [17].

Under stress conditions, including fasting and hypoxia, nutrient depletion triggers mobilization of lipids, namely FFAs, to supply energy, which involves lipid metabolism and autophagic degradation processes [21]. The TAG and CE stored in LDs can be converted in FFAs and subsequently in fatty acyl-CoA that are substrates for synthesis of complex lipids (e.g., phospholipids, CE, ceramide, and TAG) or can be used for energy production via mitochondrial β-oxidation [73]. For this process, the LDs have to be degraded by lipolysis or autophagy [21]. It has been shown that, in response to autophagy activation, sequestosome-1 (p62) acts as a selective lipophagy receptor that recruits autophagosomes to LDs [99]. Due to degradation of LDs, the autophagic process also can exert a protective effect, avoiding the toxicity of lipids released from these organelles. However, alterations in the autophagic degradation process can lead to accumulation of potentially toxic misfolded and aggregated proteins, dysfunctional mitochondria and lipids, and contribute to premature aging, tissue degeneration, and proinflammatory responses [18].

In recent years, it was demonstrated that MAMs have an important role in autophagy, especially in the autophagosome’s formation. Hamasaki et al. found that the MAMs fraction of mammalian cells is enriched in the autophagy-related proteins (ATG) 5 and ATG14, and in the FYVE domain-containing protein 1 (DFCP1) [100], suggesting that the ER–mitochondria contact is involved in the initiation of the phagophore’s expansion [101]. In HeLa cells, starvation conditions were demonstrated to increase the levels in MAMs of ATG14, which is a marker of autophagosome’s formation, while ATG5 was found to translocate to MAMs until the autophagosome is formed [100]. In the same study, it was also observed that, when ER–mitochondria contact is disrupted, the ATG14 cannot be correctly localized at MAMs and formation of autophagosomes is inhibited [100]. Moreover, it was shown that *Pacs2* (phosphofurin acidic cluster sorting protein 2) and *Mfn2* knockdown decreases the levels of ATG14, DFCP1, and microtubule-associated protein 1A/1B-light chain 3-II (LC3-II), suggesting a reduction in autophagosome’s formation due to the disruption of ER–mitochondria connectivity [100]. However, another study demonstrated that, during starvation, the number of MAMs and mitochondrial function are stimulated immediately before autophagosome’s formation. It was also observed that, when LC3-II levels increase, ER–mitochondria juxtaposition decreases, together with inhibition of mitochondrial function and upregulation of MFN2 [102]. Furthermore, knockdown of proteins involved in MAMs tethering, namely VAPB or PTPIP51, increases basal autophagy and autophagic flux, and overexpression of these proteins increases the ER–mitochondria juxtaposition while decreasing the basal autophagy and autophagic flux [103]. During the phagophore’s expansion, regulatory ATG2 protein also translocated from MAMs, where it is anchored to TOM40 and TOM70, to the phagophore [104]. These results suggest that the ER–mitochondria interaction is negatively correlated with the autophagosome’s formation [105].

Lipids also have an important role in formation of the autophagosome, especially phospholipids and sterols. The PE has an important role during expansion of the isolation membrane and binds to ATG8 (LC3) by its C-terminal glycine residue. Nair et al. observed that higher levels of PE promote connection between PE and ATG8, facilitating ATG8-mediated fusion and closure of the phagophore in vivo [106]. Furthermore, PS can also act as an ATG8 receptor [107], and LDs can be a critical source of lipids for autophagosome’s synthesis [108,109]. On the other hand, the FAs released during the autophagic process can be transferred into new LDs via the DGAT1 to prevent lipid toxicity [12]. Moreover, MAMs also control Ca^2+^-dependent autophagy via the AMPK (AMP-activated protein kinase)/mTOR (mammalian target of rapamycin)/ULK1 (unc-51-like autophagy-activating kinase 1) pathway. Upon disruption of ER–mitochondria Ca^2+^ transfer, the AMPK is translocated to MAMs and induces autophagy through Beclin1 activation [110].

Excessive accumulation of LDs was observed in autophagy-deficiency hepatocytes and MEFs when compared with control cells [85]. The autophagic process responsible for LDs degradation is called lipophagy and occurs in a variety of cells, including neurons [21]. This process can be activated under starvation or pathological conditions, and the LDs membrane proteins are recognized by autophagy-related proteins [111]. The interaction between LDs and lysosomes enables degradation via chaperone-mediated autophagy (CMA) of the structural perilipin family members perilipin 2 (PLIN2) and PLIN3 that surround the LDs [112]. In this process, PLIN2 and PLIN3 are recognized by Hsc70 (heat-shock-associated protein 70), delivered to the lysosomal surface, and translocated into the lumen through Lamp2A (lysosome-associated membrane protein) [113]. Degradation of LDs’ PLINs facilitates the association between the LDs-resident cytosolic ATGL and ATG proteins [112]. The degradation of PLINs allows the access of ATGL to LDs and facilitates LDs’ lipolysis [112]. ATGL contains an LC3 interacting region that facilitates its interaction with the LC3-containing organelles [114]. Thereafter, the LDs are engulfed by the phagosome membrane, and the autophagosome fuses with the lysosome and the degradation of LDs is catalyzed by the Aut5/Cvt17/Atg15 lipase to produce large amounts of FFAs [111]. Finally, the released FFAs can be used by mitochondria to produce ATP via β-oxidation, which plays an important role in energy recruitment during fasting conditions [109].

CMA is activated by nutritional changes such as starvation or in response to lipid overload [115]. In Lamp2A-deficient fibroblasts, an accumulation of TAG and LDs is observed when compared with control cells, as well as a reduction in fatty acid oxidation, suggesting that turnover of neutral lipids is abolished in the absence of Lamp2A [112]. Furthermore, an increased number and size of LDs and an accumulation of CE and TAG occurs in the liver from Lamp2A-knockout mouse. In addition, accumulation of TAG precursors, such as FFAs and DAG, is detected after 24 h of starvation, demonstrating an inadequate adaptation of the peripheral adipose tissue to the lipid overload [115].

Degradation of PLIN2 and PLIN3 via CMA is necessary for the association between the LDs and the cytosolic lipase and the macroautophagy effector proteins for subsequent lipolysis [112]. Accumulation of PLIN2 levels in response to impaired CMA reduces the mobilization of FAs by lipolysis and lipophagy. Furthermore, under these conditions, inhibition of LDs’ degradation and subsequent accumulation of larger and clustered LDs is observed in NIH3T3 mouse fibroblasts that also exhibit decreased interaction with lysosomes [112]. These results can indicate that formation of LD–lysosome contacts depends on the interaction between the CMA machinery and PLIN2/3 [17].

During the lipolysis process, cytoplasmic lipases ATGL, HSL, and MGL target LDs to hydrolyze FAs from TAG, DGA, and monoacylglycerol, respectively. The lipase trafficking to the LDs surface is regulated by signaling pathways involving the AMPK and the cyclic adenosine monophosphate (cAMP)/protein kinase A (PKA) [99]. Moreover, LDs-resident cofactors can also block lipase access to the LD or directly modulate the lipase activity [99].

Martinez-Lopez et al. observed that the increased autophagosome interaction with LDs is associated with the recruitment of ATGL and HSL to the LDs surface in the brown adipose tissue. Furthermore, mutations in ATGL LIR motifs (F146A, V149A) reduce LDs recruitment, preventing starvation-induced LDs loss [114]. Soni et al. suggest that autophagosomes are involved in the delivery of cytosolic lipases to the LDs surface since an association between lipase HSL and LC3 is observed upon immunoprecipitation of purified LDs [116]. In addition, the LIR motif of ATGL also appears to be critical for lipophagy, suggesting that this interaction can also promote the tethering of the autophagosome to the LDs [114]. ATGL targets larger LDs upstream of lipophagy and lipolysis, which is essential to reduce LDs size to facilitate lipophagy [117].

Recently, it was reported that *ATGL* knockdown decreases LC3-II and Lamp1 proteins levels and increases p62 levels in the mouse liver, indicating compromised autophagic flux. Furthermore, decreased colocalization between LC3 and the LDs marker PLIN2 is also observed, suggesting an impairment in lipophagy [118]. However, *Atgl* overexpression in mice liver increases the LC3-II and Lamp1 levels, LDs-LC3 colocalization, and lysosomes content, together with decreased p62 levels, which demonstrates that the autophagic flux and lipophagy are stimulated under these conditions. Similarly, *ATGL* overexpression decreases the LDs’ accumulation in lipid-loaded hepatocytes [118].

Degradation of PLIN proteins that prevents the access of cytosolic lipases to LDs appears to be stimulated by the lysosome-LDs interaction. In the CMA process, the PLIN2 and PLIN3 proteins are transported by the receptor Lamp2A in the lysosome membrane and are degraded into the lysosome lumen. Accordingly, β-oxidation due to lipolysis is found downregulated in Lamp2A-deficient cells, along with decreased ATGL trafficking to the LDs [112]. Additionally, in cells expressing a mouse PLIN2 mutant that is resistant to CMA degradation, the lipolysis and ATGL trafficking to LDs is blocked. These results suggest that LDs-lysosome “kiss and run” events can promote the degradation of LDs surface’s proteins facilitating the access to cytosolic lipases [112].

Ubiquitination of LDs-resident proteins acts as a signal for proteasome degradation, but ubiquitin modifications can also signal the recruitment of autophagosomes to the LDs. The spartin protein that is involved in hereditary spastic paraplegia localizes to LDs and recruits the E3 ubiquitin ligase AIP4 to the LDs surface [119,120]. Thereafter, spartin activates the AIP4 ligase in the LD that ubiquitinates the LD-resident protein PLIN2 [119]. Eastman et al. also showed that depletion of endogenous spartin increases the LDs’ content after oleic acid treatment, indicating that spartin/AIP4-mediated ubiquitination of PLIN2 may influence LD breakdown. On the other hand, spartin overexpression increases the content of LDs, suggesting a complex interplay between LD ubiquitination and lipid metabolism [119].

In 2013, it was demonstrated that, under starvation conditions, autophagosome formation starts at MAMs, where the specific pre-autophagosome/autophagosome markers ATG14 and ATG5 are localized [100]. The main autophagy regulator serine/threonine kinase mTOR exists in two protein complexes: the mTOR complex-1 (mTORC1) and -2 (mTORC2). The mTORC2 present at MAMs activates protein kinase B (AKT) that can control MAMs integrity and mitochondrial physiology. The complex mTORC2-AKT regulates IP3R3 phosphorylation, and consequently, the release of Ca^2+^ from the ER [28,121]. Furthermore, the Rab32 that regulates the mTORC2 activity, and is essential for autophagosome formation, is also localized at MAMs [122].

Activation of mTORC1 prevents biogenesis of LDs-induced during starvation, so LDs formation is a cellular response to periods of high autophagic flux [12]. The DGAT1 or DGAT2 inhibition alone partially blocks LDs’ biogenesis, and the inhibition of both enzymes totally prevents the formation of LDs in the presence of oleate. However, under starvation conditions, the inhibition of DGAT1, but not of DGAT2, blocks the LDs biogenesis without affecting LC3 degradation kinetics or autophagic flux [12]. In addition to DGAT1 being required for LDs formation, its inhibition decreases the basal mitochondrial oxygen consumption rate, mitochondrial membrane potential, and cellular viability during starvation. However, DGAT1 inhibition does not affect mitochondrial morphology and abundance, indicating that the alteration in mitochondria functional parameters is not due to increased mitophagy or a decreased mitochondrial biogenesis [12].

**Figure 2:**
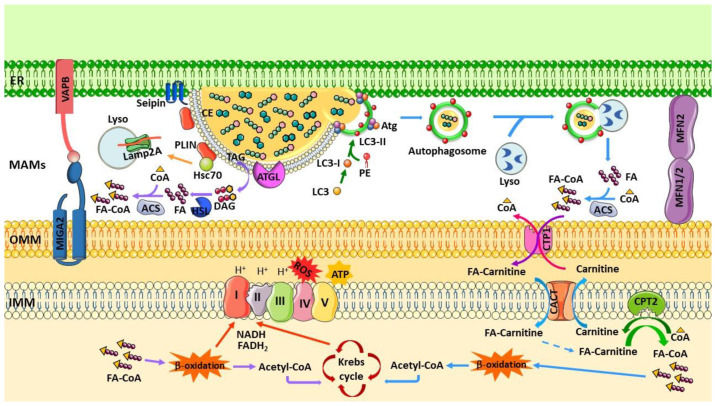
Breakdown of lipid droplets by lipolysis and lipophagy. ER and mitochondria can interact through complexes VAPB-Miga2 and MFN2-MFN1/2. Under nutrient depletion or cell growth, the core of LDs can be mobilized by lipolysis or lipophagy. Hsc70 recognizes LDs-associated proteins PLIN and translocates this protein into the lysosome via Lamp2A for degradation, facilitating access of the lipid core to cytosolic lipase ATGL. TAG is converted to DAG by ATGL and DAG to FAs by HSL protein. Similarly, elimination of PLINs also enables proteins involved in autophagosome formation (Atg and LC3) to anchor on LDs and initiate sequestration of regions of LDs in autophagosomes. Autophagosomes are delivered to lysosomes for degradation by acid lipolysis. FAs released are converted in fatty acyl-CoA by acetyl-CoA synthetase (ACS) and transferred to the mitochondrial matrix by a carnitine shuttle constituted by CPT1, CACT, and CTP2. In mitochondria, β-oxidation of fatty acyl-CoA occurs, leading to production of acetyl-CoA that enters the Krebs cycle. NADH and FADH_2_ generated both from β-oxidation and the Krebs cycle are the electron donors for the electron transport chain for ATP production, which is also associated with generation of ROS.Abbreviations: ACS, acetyl-CoA synthetase; ATG, autophagy-related protein; ATGL, adipose triglyceride lipase; CACT, carnitine-acylcarnitine translocase; CE, cholesteryl esters; CoA, coenzyme A; CPT1/2, carnitine-palmitoyltransferase 1/2; DAG, diacylglycerol; ER, endoplasmic reticulum; FA, fatty acid; FA-CoA, fatty acyl CoA; Hsc70, heat-shock-associated protein; HSL, hormone-sensitive lipase; IMM, inner mitochondrial membrane; Lamp2A; lysosome-associated membrane protein 2A; LC3, microtubule-associated protein 1A/1B-light chain 3; LD, lipid droplets; Lyso, lysosome; MAMs, mitochondria-associated membranes; MFN1/2, mitofusin 1/2; Miga2, mitoguardin 2; OMM, outer mitochondrial membrane; PE, phosphatidylethalonamine; PLIN, perilipin; TAG, triacylglycerol; VAPB, vesicle-associated membrane-protein-associated protein B.

The pathogenic deposition of lipids is involved in the physiopathology of many disorders, including neurodegenerative diseases, and stimulation of lipophagy or lipolysis is essential to reduce the accumulation of lipids maintaining lipid homeostasis through the regulation of lipid stores [123] (Figure 2).

## 5. Lipid Droplets and Neurodegenerative Diseases

Lipids are essential structural and functional components of the brain, which is rich in long-chain PUFAs, including arachidonic acid (AA), eicosapentaenoic acid, and docosahexaenoic acid (DHA) [19]. Despite some FAs can be synthesized de novo, essential FAs are transported from the systemic circulation into the brain by diffusion across the blood-brain barrier (BBB), followed by uptake into neurons through FA transporters [19]. Once transported into neurons, long-chain FA are esterified by ACSL to long-chain FA-CoA [124]. Consequently, the inhibition of ACSL disrupts the accumulation of long-chain FA-CoA, as observed in the hypothalamus [125].

Changes in lipid storage are related with several pathological conditions, including obesity, inflammation, atherosclerosis, cancer, and neurodegenerative diseases [21]. Insufficient lipid storage can lead to lipotoxicity, mitochondrial dysfunction, and increased oxidative stress. However, the excessive lipid accumulation leads to hypoxia, ER stress, immune cell infiltration, and increased secretion of proinflammatory cytokines [126].

Since the brain is the second most lipid-rich organ, alterations in lipid composition of the CNS affect cell function and normal neural activity [20]. The levels of TAG are low in neurons and evidence that LDs are formed in neurons in vivo is limited [127,128], since these cells are constantly turning over TAG to synthesize phospholipids for membranes [127,129]. However, in cultured neurons from the hippocampus [130], dorsal root ganglion [127], striatum [131], and hypothalamus [132], LDs are frequently detected.

The increase in the number of LDs was induced by cellular stress, including excitotoxicity [130] or treatment with FAs [130,132]. Furthermore, the accumulation of LDs can be caused by increased oxidative stress, because both intracellular ROS or exposure to oxidative stressors, such as hydrogen peroxide, induce LDs formation [133,134,135]. During nutrient depletion, intracellular LDs are degraded by autophagy and the resulting FAs are used by mitochondria as an alternative source of fuel [81]. However, neurons have a low capacity to use FAs for ATP production [136], making neurons particularly sensitive to LDs accumulation and to increased FAs oxidation, which contribute to enhanced production of ROS by mitochondrial electron transport chain complexes I and III exacerbating oxidative stress [22]. However, ROS production by extra-mitochondrial sources, including NADPH oxidases, seems to be also associated with lipid overload. The low capacity of neurons for FAs metabolization associated with the decreased levels of antioxidant defenses contribute to LDs accumulation and, consequently, to oxidative stress, mitochondrial damage, lysosomal dysfunction, defective autophagy, and activation of inflammatory responses through the secretion of proinflammatory cytokines [23,137].

Accumulation of LDs and their peroxidation can accelerate intracellular organelles stress contributing to the development of several pathologies including neurodegenerative diseases [23]. It has been reported that autophagy can have an essential role in maintaining LDs homeostasis [23,138], since they can be sequestered in autophagosomes and delivered to lysosomes for degradation by lipophagy, the autophagic degradation of LDs [139].

In recent years, impairment of autophagy and abnormal intracellular accumulation of LDs have been described as pathological hallmarks in neurodegenerative and neuroinflammatory disorders [84]. Furthermore, dysfunction of MAMs and lipid dyshomeostasis are important risk factors in neurodegenerative diseases, including Alzheimer’s disease (AD), amyotrophic lateral sclerosis (ALS), and Parkinson’s disease (PD) [111].

### 5.1. Alzheimer’s Disease

AD is a neurodegenerative disorder characterized by the accumulation and deposition of extracellular β-amyloid (Aβ), named senile plaques, and intracellular hyperphosphorylated tau, called neurofibrillary tangles and progressive neuronal loss, especially in the cortex and hippocampus, among others [140] (Table 1). Alterations in lipid metabolism were observed in AD, a pathological condition where the autophagy-lysosomal pathway plays a critical role in intracellular clearance of misfolded and aggregated proteins and metabolic homeostasis [18]. The accumulation of LDs characterized by high concentrations of oleic acid-enriched TAG was reported in the 3xTg-AD mouse model and AD human *postmortem* brain tissue within ependymal cells, the main supporting cells found in the forebrain neural stem cells (NSC) niche. Furthermore, intracerebroventricular (ICV) infusion of oleic acid in wild-type (WT) mice was sufficient to recapitulate AD-associated ependymal TAG phenotype and to inhibit NSC proliferation. However, the inhibition of stearoyl-CoA desaturase, the rate-limiting enzyme of oleic acid synthesis, recovered NSC proliferation defects in 3×Tg-AD mice [141]. Other study also demonstrates the increase of LDs number in fibroblasts from familial AD (FAD) and sporadic AD (SAD) patients when compared with controls [48]. Mutations in the genes that encode constituents of the catalytic core of γ-secretase complex, both presenilin-1 (PS1) and presenilin-2 (PS2), which cause early onset FAD, are associated with alterations in MAMs formation and lipid metabolism dysregulation [142]. The increase of cholesteryl esterification and LDs was observed in PS KO MEFs cells followed by the increase of MAMs formation [48]. Moreover, the increase of LDs was also observed in PS1 -mutant MEFs and in knockdown PS1 (PS1-KD) cells and the overexpression of WT PS1 decreased the LDs number in PS1-KD cells [48]. Area-Gomez et al. also reported that PS2 contributes to phospholipid metabolism and MAMs function, since lipid synthesis in the PS1 + PS2 double KO was more pronounced than in the PS1-KO alone [48]. Furthermore, the lack of MFN2 in MEFs, a protein involved in ER–mitochondria tethering, leads to a decrease of CE and phospholipid synthesis [48]. Moreover, increases of LDs levels were found in fibroblasts from FAD patients with mutations in PS1, PS2, and APP, and in SAD fibroblasts when compared with controls [48]. The levels of lipids stored in LDs was also reported to be increased in AD. The elevated levels of DAGs were also found in plasma [143,144] and frontal cortex [145,146] of AD patients as well as in temporal cortex of mixed dementia patients with AD and subcortical ischemic vascular dementia [147], suggesting that DAG can be involved in AD pathophysiology. The levels of the storage form of cholesterol, CE, are increased in brain samples of late onset of AD (LOAD) [145], in brain from transgenic PS1-APP [148] and APP/tau [145] mice, and in AD patient-derived neurons [149]. The CE potentiates the accumulation of phosphorylated tau (p-tau) by decreasing the proteosome activity and the reduction of CE concentration with both statins and an allosteric activator of cholesterol 24-hydroxylase (efavirenz) lowered p-tau levels in human neurons [149]. An increase in MAMs ACAT1 protein levels that are involved in cholesterol esterification was also observed in the hippocampus of APP (Aβ precursor protein)/PS mice and in SH-SY5Y cells exposed to Aβ_25–35_ peptide [150], increasing the CE production. Puglielli et al. reported in CHO cells and in a neuronal cell model that CE levels were correlated with Aβ production, and also that the inhibition of ACAT decreased the CE content and consequently, reduced Aβ generation [151]. Other studies also showed that, ACAT1 silencing attenuated Aβ-induced cytotoxicity and cell apoptosis in SH-SY5Y cells due to the activation of protein kinase C (PKC) and extracellular-signal-regulated kinase (ERK) [150]. Moreover, other studies showed that the inhibition of ACAT1 enzyme decreased the amount of Aβ secreted into the growth medium in cells expressing human APP (hAPP) [151]. Transgenic mice expressing human APP (751) containing the London (V717I) and Swedish (K670M/N671L) mutations treated with isotype-nonspecific ACAT inhibitors, CP-113,818 and CI-1011 showed a reduction of Aβ and amyloid plaque levels and an amelioration of cognitive deficits [152,153]. Similar observations were made in the 3×Tg-AD mouse model, in which *Acat1* gene knockout results in decreased hAPP and Aβ_1-42_ levels and rescue of cognitive deficits [154,155]. Moreover, knockdown of *Acat1* gene in symptomatic 3×Tg-AD mice decreases the hAPP and Aβ_1–42_ levels [156]. These observations can result, at least partially, from autophagy stimulation, since it was reported that in primary microglia isolated from neonatal mouse brains and in murine microglial cell line N9 the blockage of ACAT1 by genetic inactivation or by using K604, a potent ACAT1-specific inhibitor, increased autophagosomes formation, stimulated lysosomal proteolysis, and facilitated Aβ_1-42_ peptide degradation [157]. The blockage of ACAT1 in in vitro and in vivo models that express human tau also increases autophagosomes formation and decreases P301L-tau protein levels [158].

The deletion of *Atg7,* a key mediator of autophagosome biogenesis, in the microglia cell line (BV2) not only promoted a proinflammatory response and inflammasome activation, but also caused the accumulation of LDs [18]. Furthermore, in *Atg7-*KO BV2 cells the activation of apolipoprotein E (ApoE) and lipid efflux attenuates LDs accumulation and inhibits cytokine production [18]. The increase of p-tau levels and neurofibrillary tangles pathology was also observed in microglial *Atg7* deficient PS19 mice. These observations revealed that autophagy plays an important role in lipid homeostasis, neuroinflammation, and tau pathology in microglia [18].

In human induced pluripotent stem cells (iPSC)-derived astrocytes and in choroid plexus of *postmortem* mice and human brain tissues, the accumulation of LDs was associated with ApoE4 [141], which is present at MAMs, and was reported to upregulate the phospholipid transport/synthesis in this region in human fibroblasts and primary mouse hippocampal neurons [159]. Ioannou et al. demonstrated that toxic FAs produced in hyperactive neurons or neurons exposed to oxidative stress are transported to astrocytic LDs by ApoE-positive lipid particles and in astrocytes FAs stored in LDs are consumed via mitochondrial β-oxidation, protecting neurons from FAs toxicity during periods of high activity [130]. In astrocytes, LDs accumulation can protect from ROS-mediated stress by modulation of antioxidant defense mechanisms and apoptotic signaling [160]. The LDs accumulation mediated, in part, by ApoE4 was also observed in microglia of fly models of AD [161]. Initially, the storage of peroxidized lipids in microglia can have a protective effect but the accumulation of LDs in these cells can induce oxidative stress and inflammation [84]. In addition, LDs accumulation can suppress the homeostatic and regenerative functions of neural stem cells in the 3×Tg-AD mouse model of AD [141].

Overexpression of ApoE4 affects lipid metabolism in astrocytes, increasing the utilization of endogenous FAs and LDs formation [162]. The transport of toxic peroxidized FAs from hyperactive neurons to astrocytes LDs is mediated by ApoE. After, astrocytes consume FAs stored in LDs by β-oxidation [130,163]. However, ApoE4 is a strongest genetic risk factor for the LOAD and overexpression of this ApoE isoform in neurons and astrocytes decrease the storage of neuronal FAs into neuronal LDs, reduce the transfer of lipids between neurons and astrocytes, and, consequently, decrease FAs β-oxidation. This result in lipid accumulation and affect the neuronal function [164].

In 5×FAD and 3×Tg-AD mice, the increase of p62 or transcriptional factor EB (TFEB) activity attenuates Aβ plaque formation [165]. The levels of autophagy-related proteins are commonly altered in AD patients’ samples [166,167,168]. Genetic and histological evidence demonstrates disturbed autophagy, and lipophagy plays an important role in amyloid and tau pathology in AD [84]. A gene associated with a high risk of developing LOAD, *PICALM* (phosphatidylinositol binding clathrin assembly protein), is related with the assembly and maturation of autophagosomes [161]. The accumulation of autophagosomes has been linked to failure in clearing accumulated Aβ and tau proteins [84]. Furthermore, autophagosome-lysosome fusion inhibition by chloroquine affects tau clearance and causes the accumulation of tau aggregates [169].

The accumulation of cholesterol and ceramide species contributes to AD pathophysiology and the inhibition of cholesterol accumulation prevents neuronal death [170]. The downregulation of autophagy was observed in AD brains associated with the accumulation of immature autophagic vacuoles in dystrophic neurites of AD human subjects, suggesting the impairment of autophagic vacuoles trafficking or autophagosome-lysosome fusion [171]. Furthermore, decreased levels of the autophagy players Beclin1 and VPS35 (vacuolar protein sorting ortholog 35) are found in AD brains [172,173]. In mice, generic downregulation of Beclin1 caused the decrease of neuronal autophagy and Aβ accumulation contributing to neurodegeneration [172]. The Beclin1 promote APP trafficking to the autophagosomes, facilitating its proteolytic cleavage [174,175] and modulates phagocytosis in neurons by regulating VPS35 protein levels [173]. More, in culture cells, knockdown of VPS35 caused Aβ accumulation [176]. Moreover, in *postmortem* AD brains, the phosphorylated tau was found to co-localize with LC3 and p62 proteins, suggesting that tau proteins are engulfed into autophagosomes, but are not degraded by autolysosomes [177]. Several studies show that the activation of autophagy-lysosomal pathway attenuates AD pathology [178,179,180]. Xiao et al. demonstrated both in vitro and in vivo AD models that exogenous expression of the lysosomal regulator TFEB stimulates lysosomal biogenesis accelerating APP degradation and, consequently, decreasing Aβ generation and amyloid plaques [178]. A previous study also showed that in a tau transgenic mouse model the TFEB increases the degradation of only aberrant hyperphosphorylated and misfolded tau and rescues neurodegenerative and behavioral deficits [179]. Furthermore, the decrease of *NRBF2* and *NRBF2*-associated autophagy complex was found in *postmortem* brain of AD subjects. Deletion of *NRBF2* impaired memory in mice, affected long-term potentiation, decreased autophagy in mouse hippocampus and promoted accumulation of APP C-terminal fragments and Aβ. However, the *NRBF2* overexpression in mouse hippocampus rescues the impaired autophagy and memory deficits of *NRBF2*-KO mice, but also decreases Aβ levels and improves memory in 5×FAD mice [180].

Since LDs accumulation plays a key role in AD pathology, lipid metabolism and LDs formation could represent important therapeutic targets in AD. Moreover, the modulation of lipophagy could also represent a potential therapeutic strategy to decrease the accumulation of LDs in AD [84].

### 5.2. Amyotrophic Lateral Sclerosis

Age-dependent neurodegenerative disease ALS is caused by the degeneration of motor neurons, commonly associated to protein inclusions resulting from pathogenic variants in four main genes: superoxide dismutase 1 (SOD1), TAR DNA-binding protein 43 (TDP-43), RNA-binding protein FUS, and C9orf72 [84,181] (Table 1). Defects in genes associated with autophagy was also associated with the disease, such as selective autophagic receptors (p62 and OPTN), autophagosome maturation (VCP), and regulators of autophagic flux (ubiquilin 2 and TBK1) [84,182]. Neurons from C9ALS/FTD patients show decreased levels of autophagy and the reduction of C9orf72 expression in culture neurons attenuates autophagy and promotes the accumulation of intracellular p62 puncta suggesting protein accumulation [183].

Recently, evidence of abnormal lipid metabolism and LDs accumulation has been observed in ALS. Mutations in genes involved in LDs biogenesis and dynamics modulate the ALS phenotype in flies, worms, and mice [184]. Mutations in the human VAPB (hVAPB) is an important risk factor for ALS and deficiency of VAPB causes LDs accumulation and clustering in muscles, which may constitute a compensatory mechanism counterbalancing skeletal muscle mitochondrial dysfunction in *C. elegans*, since the TAG accumulation in muscle helps to maintain the ATP levels via β-oxidation [185]. In *Drosophila* acyl-CoA synthetase long-chain (FACL/ACSL) promote LDs biogenesis and contributes to hVAPB-induced neurotoxicity [184]. The downregulation of FACL/ACSL levels decreases LDs nucleation reducing the size and number of mature LDs [186]. Moreover, mutations in the protein seipin, enriched at MAMs, led to motor neuron disease symptoms in mice [184,187] indicating that the impairment of LDs biogenesis can play an important role in the pathological aspect of hVAPB-mediated ALS [20]. Mutations in *SPG11* gene are related with the early onset form of ALS and affect lysosomes recycling, inducing intracellular lipid accumulation [188]. Branchu et al. observed that *Spg11* knockout mice presented a slower rate of lipid clearance and a decrease in LDs size and number when compared with WT mice [188]. Furthermore, knockout of *C9orf72* gene in MEFs has led to cellular hypermetabolism, as a response to glucose starvation, with the increase of de novo FAs synthesis and LDs number mediated by dysregulation of autophagic digestion of lipids [189]. The hexanucleotide repeat expansion in *C9orf72 gene* is the most common cause of ALS [190] and C9orf72 promotes the lysosomal degradation of the coactivator-associated arginine methyltransferase 1 (CARM1), which regulates autophagy-lysosomal functions and lipid metabolism downregulating autophagy [189,191]. It was also found that a decrease of C9orf72 function is associated with a dysregulation in CARM1 protein levels, FFAs concentration, and NADPH oxidase NOX2 protein levels in *C9orf72* knockout (C9KO) MEFs and ALS/FTD (frontotemporal dementia) motor neurons derived from human iPSCs and spinal cord tissues [189]. In iPSC-derived motor neurons from C9orf72-linked ALS patients the reduced lysosomal LDs clearance increases oxidative stress contributing to neuronal damage and neurodegeneration [189]. Other studies showed a correlation between LDs and cellular stress. Bailey et al. observed that ROS accumulation increases the glial LDs content in *Drosophila* and the peroxidative damage of neuroblasts increases when glia are unable to produce LDs [97]. Moreover, Simpson et al. found a positive correlation between lipid peroxidation markers in ALS patient cerebrospinal fluid and disease burden [192].

These observations in ALS pathology indicate that dysregulation of autophagy and lipid metabolism, including alterations in LDs dynamics, can contribute to disease progression [84].

### 5.3. Parkinson’s Disease

PD is characterized by the presence of Lewy bodies containing aggregates of α-synuclein protein that led to *substantia nigra* dopaminergic neurons loss, contributing to abnormal brain activity and development of several symptoms, including tremors, bradykinesia, and limb rigidity [193]. Several processes have been implicated in PD pathogenesis, including mitochondrial dysfunction, dopamine dyshomeostasis, neuroinflammation, and autophagy inhibition [194] (Table 1). Impairment of the autophagy pathway has been related to α-synuclein accumulation and it has been shown that this protein plays a role in the initial phase of phagosome formation [195]. Furthermore, it was reported that WT α-synuclein from cells and brain tissues was located at MAMs and pathogenic point mutations in human α-synuclein were shown to reduce its association with MAMs and, consequently, decrease ER–mitochondria tethering and phospholipid synthesis and increase mitochondrial fragmentation when compared with WT α-synuclein M17 neuroblastoma cell line [196]. Inhibition of CMA or deletion of CMA recognition motif in the gene that encodes for α-synuclein can exacerbate its aggregation [197]. Decreased expression of CMA core proteins Lamp2A and Hspa8 in blood samples from PD patients indicates that CMA is impaired in early stages of PD [198]. Mutations in *PINK* and *Parkin* genes that encode for selective-autophagy receptors, mainly targeting defective mitochondria, are associated with PD development [199]. In addition, alterations in leucine-rich repeat kinase (LRRK2), which is associated with phagosome assembly, also play a major role in PD [200]. Additionally, impairment of autophagy and failure to degrade protein aggregates were also observed in brain tissues of PD animal models and human subjects [195]. Gelmetti et al. showed that, following mitophagy stimulus, endogenous PINK1 (PTEN-induced kinase 1) and Beclin1 relocalize at MAMs and enhance ER–mitochondria contacts sites and formation of autophagosomes precursors. Levels of PARK2 (parkin2) at MAMs also increase after mitophagy induction [201].

Mechanisms involved in PD pathogenesis, such as oxidative stress, lysosomal dysfunction, ER stress response, and immune response, are dependent on genes involved in lipid and lipoprotein signaling [20]. Furthermore, α-synuclein toxicity and alterations in cell trafficking have been associated with defects in LDs content and distribution. Although PD has been classically associated with “proteinopathy”, recent studies show that lipid dyshomeostasis is one of the fundamental characteristics of this disorder [202] and that LDs-related lipotoxicity seems to be involved in PD pathology [203]. In fact, in *postmortem* brain tissue from PD subjects, a decrease was found in neutral lipids content in astrocytes present in the substantia nigra, while their content in neurons and microglia increased, indicating dysregulation of lipid homeostasis is this disorder [204].

Accumulation of LDs was observed in the brain of methyl-4-phenyl-1,2,3,6-tetrahydropyridine (MPTP) neurotoxin-induced PD mice, suggesting that these cellular components can be involved in PD pathogenesis [205]. Alterations in the number and distribution of LDs have been associated with α-synuclein toxicity. The α-synuclein protein can accumulate in LDs phospholipid surfaces, slowing their lipolysis [206]. In primary hippocampal neurons from mice, LDs serve as a platform for WT α-synuclein accumulation on its surface [206]. Accumulation of α-synuclein on LDs surface protects TAG stored from degradation, causing LDs accumulation in yeast cells [207]. The α-synuclein mutants, namely E46K and KTKEGV, have high affinity to LDs and cause protein aggregation in the proximity of LDs [206]. Decreased capacity of LDs turnover in neurons was also observed in two mutant forms of α-synuclein, A30P and A53T, when compared to WT α-synuclein [206]. Furthermore, accumulation of LDs in yeast was also found to be associated with WT and A53T α-synuclein, whereas A30P α-synuclein was not, suggesting that LDs may play a cell-specific role in PD pathology [207]. The two mutants of α-synuclein (A30P and A53T) are related with rare forms of early-onset familial PD but have different physical properties. WT and A53T α-synuclein are concentrated at the plasma membrane, whereas A30P α-synuclein is dispersed throughout the cytoplasm and affects yeast cell growth. Alterations in genes related to lipid metabolism and vesicle-mediated transport increase α-synuclein toxicity in yeast [208]. In a *Drosophila* model, it was observed that lipid-related genes are associated with α-synuclein expression, and dysregulation in lipid processing may be caused by A30P α-synuclein toxicity [209]. In N27 dopaminergic neuronal cell line, overexpression of A53T α-synuclein triggered an increase in TAG levels due to activation of ACSL present at MAMs [210].

In yeast, inhibition of oleic-acid-generating enzyme stearoyl-CoA-desaturase (SCD) had a protective role against α-synuclein toxicity, and SCD knockout models prevent dopaminergic neurons degeneration [202]. Moreover, mutations in ATPase cation transporting protein 13A2 (ATP13A2), involved in lipid regulation, are associated with PD, and in vitro overexpression of this protein decreases several lipid species [211]. Furthermore, α-synuclein reduces autophagic activity during maturation of autophagosomes and their fusion with lysosomes [212]. Mutations in ATP13A2 lead to autophagosomes accumulation since they affect lysosomal pH and autophagosome–lysosome fusion [213].

MPTP neurotoxin-induced PD mice present elevated levels of LDs accumulation, increased loss of dopaminergic neurons, ROS-mediated stress, and behavioral deficits [203]. However, natural flavonoid kaempferol (Ka) that has antioxidant and neuroprotective effects [214,215] prevents LDs toxicity in vitro and ameliorates dopaminergic neuronal loss and behavioral deficits in the MPTP neurotoxin-induced PD mice. [203]. All these effects were autophagy-dependent since Ka can activate this quality control mechanism. Thus, enhanced autophagy decreases accumulation of oxidized LDs, reduces mitochondrial ROS production, and alleviates mitochondrial dysfunction in dopaminergic neurons, preventing neuronal apoptotic death [203].

Proteins involved in lipid homeostasis and LDs biology have been shown to be associated with PD [216]. Alterations in genes related with lipid accumulation, including *DGKQ* (diacylglycerol kinase), which controls DAG levels, and *ELOVL7* (FA elongase 7), involved in fatty acyl-chain length, are PD risk factors. Additionally, mutations in the *BSCL2* (Berardinelli–Seip congenital lipodystrophy syndrome 2) gene that encodes for MAMs seipin protein that is involved in LDs formation has also emerged as a PD risk factor [217]. Moreover, it was observed that neuronal *seipin* knockout in mice enhanced aggregation and phosphorylation of α-synuclein and led to neuroinflammation, and, consequently, causes loss of dopaminergic neurons [218].

Genetic alterations in the leucine-rich repeat kinase 2 (*LRRK2*) gene that are associated with both familial and sporadic PD [219] and changes in lipid homeostasis were observed in LRRK2^−/−^ mice, including increases in LDs formation when compared with WT animals [220]. Accumulation of LDs was also observed in human iPSC-derived dopaminergic neurons from LRRK2 G2019S mutation carriers [221]. Moreover, LRRK2 substrate Rab8a plays a role in LDs fusion and enlargement [222,223] and the co-overexpression of LRRK2 (Y1699C) and Rab8a increases LDs formation when compared with Rab8a overexpression alone in 3T3-L1 pre-adipocytes cells [222]. Furthermore, mutations in LRRK2 affect lysosomal function, with increases in lysosome size and reduction in the number of lysosomes [224], as well as affect lysosomal lipid BMP (lysobisphosphaditic acid) [225], which plays a role in formation, structure, and trafficking of endolysosomes [226]. LRRK2 substrates Rab5 and Rab10 are also involved in recruiting endosomes to LDs [227] and recruiting the autophagy machinery for LDs engulfment, respectively [228]. Therefore, alteration in LRRK2, which regulates ER–mitochondria tethering [229], affects lipids and proteins involved in lipid metabolism.

Recently, Alarcon-Gil et al. reported that linoleic acid has a neuroprotective and anti-inflammatory role in in vitro and in vivo models of PD through increase in LDs formation, which was shown to be dependent on MAMs DGAT2 enzyme activation and improvement in autophagy/lipophagy flux, which resulted in a protective effect [230]. An increase in DAG levels was observed in frontal cortex from PD patients [231] and interfering with TAG/LD biosynthesis via DGAT through deletion of DGAT ortholog in yeast or depletion of DGAT1 and 2 in rodent cortical neurons potentiate α-synuclein toxicity [202,232]. Furthermore, inhibition of autophagic flux with chloroquine increased peroxidation of LDs [230]. These results show that (1) induction of LDs biogenesis by linoleic acid is important for storage of LA as TAG in LDs and, consequently, acts as an oxidant scavenger, and (2) stimulation of lipophagic flux by LA enables removing accumulated peroxidized LDs [230].

These results show that autophagy impairment and lipid dyshomeostasis, including LDs accumulation, play an important role in PD pathology [84].

**Table 1:** Description of neurodegenerative disorders, AD, ALS, and PD.

Disease	Etiology	Neuropathology	Molecular Pathway	Pharmacological Therapies	References
AD	APP; PS1/PS2; ApoE4	Accumulation of Aβ peptide in senile plaques; Deposition of neurofibrillary tangles; Synaptic loss; Neuronal degeneration	Impaired lipid and glucose metabolism; Alterations in Ca^2+^ homeostasis; ER stress; Mitochondrial dysfunction; Oxidative stress	Cholinesterase inhibitors; N-methyl-d-aspartate receptor antagonist memantine; Aducanumab	[233,234,235,236,237]
ALS	SOD1; TDP-43; FUS; C9orf72	Degeneration of motor neurons; Loss of myelinated axons in the lateral and anterior columns of the spinal cord; Decrease in size of anterior horn of the spinal cord; Neuroinflammation; TDP-43 ubiquitin-positive inclusions	Increased glutamate-mediated excitotoxicity; Increased apoptosis; Defective axonal transport; Oxidative stress; Mitochondrial impairment; Unregulated immune responses; Accumulation of misfolded proteins; Autophagy dysregulation	Glutamate inhibitor; GABA agonist; Antioxidant Edaravone; Antisense nucleotides (ASOs); Benzodiazepine; Alpha-2 adrenergic receptor agonist	[84,181,182,183,238,239,240,241,242,243]
PD	PARK2; PINK1; LRRK2; α-synuclein	Accumulation of α-synuclein; Neuroinflammation; Loss of *substancia nigra* dopaminergic neuros	Mitochondrial dysfunction; ER stress; Oxidative stress; Dopamine dyshomeostasis; Inflammation; Autophagy inhibition	Dopamine agonist; MAO-B inhibitors; COMT inhibitors; Anticholinergic drugs	[193,194,199,200,244,245]

## 6. Conclusions

In recent years, MAMs have moved from a biochemical phenomenon to center stage of cell biology due to their role in several important processes of cellular physiology. Disruption in communication between organelles, such as mitochondria and ER, which, among other alterations, affects lipid metabolism, plays an important role in several neurodegenerative diseases.

Accumulation of LDs has been associated with neurodegeneration and aberrant cerebral metabolism, among other disorders. Therefore, LDs represent potential biomarkers and therapeutic targets. Restoring lipid balance, decreasing LDs levels, or improving some lipid metabolic pathways could represent novel approaches to treat neurodegenerative disorders. Moreover, activation of lipophagy can ameliorate neurodegenerative diseases by decreasing lipotoxicity, mitochondrial dysfunction, and ROS production. However, despite storage of FFA inside LDs being able to protect cells against stress caused by lipotoxicity, exposure to free radicals, and nutrient deprivation, LDs accumulation can lead to neurodegeneration and contribute to development of CNS diseases.

This review supports the growing research interest in lipid (dys)metabolism as an important player in neurodegeneration and the understanding that various neurological pathologies are not only proteinopathies but also lipidopathies.

## Data Availability

Not applicable.

## Abbreviations

Aβ, amyloid-β; ACAT1/SOAT1, acyl-CoA, cholesterol acyltransferase/sterol O-acyltransferase 1; ACS, acetyl-CoA synthetase; AD, Alzheimer’s disease, AKT, protein kinase B; ALS, amyotrophic lateral sclerosis; AMPK, AMP-activated protein kinase; ApoE, apolipoprotein E, APP, Aβ protein precursor; ATG, autophagy-related protein; ATGL, adipose triglyceride lipase; ATP13A2, ATPase cation transporting protein 13A2; BAP31, B-cell-receptor-associated protein 31; BiP, binding immunoglobulin protein; BMP, lysobisphosphaditic acid; cAMP, cyclic adenosine monophosphate; CARM1, coactivator-associated arginine methyltransferase 1; CE, cholesteryl esters; CerS, ceramide synthase; CMA, chaperone-mediated autophagy; CNS, central nervous system; COPII, cytoplasmic coat protein complex II; DES, dihydroceramide desaturase; DFCP1, FYVE domain-containing protein 1; DGAT1/2, diacylglycerol O-acyltransferase 1/2; ER, endoplasmic reticulum; ERK, extracellular-signal-regulated kinase; ERLIN1/2, ER-lipid-raft-associated protein 1/2; FAs, fatty acids; FACL/ACSL, fatty acid CoA ligase; FAD, familial AD; FFAs, free FAs; FIS1, mitochondrial fission 1; FTD, frontotemporal dementia; GRP75, glucose regulated protein 75; hAPP, human APP; Hsc70, heat-shock-associated protein 70; HSL, hormone-sensitive lipase; IMM, inner mitochondrial membrane; Insig 1, insulin-induced gene 1; IP3R, inositol 1,4,5 trisphosphate receptor; iPSC, induced pluripotent stem cells; Ka, kaempferol; Lamp; lysosome-associated membrane protein; LC3, microtubule-associated protein 1A/1B-light chain 3; LDs, lipid droplets; LOAD, late onset of AD; LRRK2, leucine-rich repeat kinase; MAMs, mitochondria-associated membranes; MCU, mitochondrial calcium uniporter protein; MEFs, mouse embryonic fibroblasts; MFN1/2, mitofusin 1/2; MGL, monoacylglycerol lipase; MIGA2, mitoguardin 2; mTORC1/2; mammalian target of rapamycin (mTOR) complex-1/2; MPTP, methyl-4-phenyl-1,2,3,6-tetrahydropyridine; NADPH, nicotinamide adenine dinucleotide phosphate; NRBF2, nuclear receptor binding factor 2; NSC, neural stem cell; OMM, outer mitochondrial membrane; OPTN, optineurin; p62, sequestosome-1; PACS2, phosphofurin acidic cluster sorting protein 2; PARK2, parkin2; PC, phosphatidylcholine; PD, Parkinson’s disease; PE, phosphatidylethalonamine; PEMT, PE N-methyltransferase; PICALM, phosphatidylinositol binding clathrin assembly protein; PINK1, PTEN-induced kinase 1; PKA/C, protein kinase A/C; PLIN, perilipin; PS1/2, presenilin 1/2; PS, phosphatidylserine; PSS1/2, PS synthases 1/2; PSD, PS decarboxylase; PTPIP51, protein tyrosine phosphatase interacting protein 51; PUFAs, polyunsaturated FAs; TAG, triacylglycerol; TFEB, transcriptional factor EB; TOM40/70, translocase of the outer mitochondrial membrane 40/70; ROS, reactive oxygen species; SAD, sporadic AD; Scap, SREBP cleavage-activating protein; SCD, stearoyl-CoA-desaturase; SERCA, sarco/endoplasmic reticulum Ca^2+^ ATPase; Sigma1R, sigma 1 receptor; SMase, sphingomyelin kphosphodiesterase; SREBP, sterol-regulatory-element-binding protein; ULK-1, unc-51-like autophagy-activating kinase 1; UPR, unfolded protein response; VAPA/B, vesicle-associated membrane-protein-associated protein A/B; VDAC, voltage-dependent anion channel; VPS35, vacuolar protein sorting ortholog 35; WT, wild-type.
